# Computational Modeling of Microabscess Formation

**DOI:** 10.1155/2012/736394

**Published:** 2012-11-08

**Authors:** Alexandre Bittencourt Pigozzo, Gilson Costa Macedo, Rodrigo Weber dos Santos, Marcelo Lobosco

**Affiliations:** ^1^Graduate Program in Computational Modeling, UFJF, Rua José Lourenço Kelmer s/n, Campus Universitário, Bairro São Pedro, 36036-900 Juiz de Fora, MG, Brazil; ^2^Graduate Program in Biological Sciences, UFJF, Rua José Lourenço Kelmer s/n, Campus Universitário, Bairro São Pedro, 36036-900 Juiz de Fora, MG, Brazil

## Abstract

Bacterial infections can be of two types: acute or chronic. The chronic bacterial infections are characterized by being a large bacterial infection and/or an infection where the bacteria grows rapidly. In these cases, the immune response is not capable of completely eliminating the infection which may lead to the formation of a pattern known as microabscess (or abscess). The microabscess is characterized by an area comprising fluids, bacteria, immune cells (mainly neutrophils), and many types of dead cells. This distinct pattern of formation can only be numerically reproduced and studied by models that capture the spatiotemporal dynamics of the human immune system (HIS). In this context, our work aims to develop and implement an initial computational model to study the process of microabscess formation during a bacterial infection.

## 1. Introduction

The immune system is one of the most important and complex system of our organism. Despite great advances in recent years that shed light on its understanding and unravel the underlying key mechanisms behind its functions, there are still many functions of the human immune system (HIS) that are not well understood. Computational models of HIS dynamics can contribute to a better understanding of the relationship between cells and molecules of the HIS.

In this study, we developed a mathematical model of some cells and molecules of the HIS to reproduce the spatiotemporal dynamics of the initial formation of microabscesses during an immune response to a bacteria.

To reproduce these dynamics, we introduce a mathematical model composed of a system of partial differential equations (PDEs) that extends our previous models [1, 2] and defines the dynamics of representative cells and molecules of the HIS during the immune response to a bacteria. The model presented is descriptive, mechanistic, and deterministic; therefore, it enables the understanding of how different complex phenomena, structures, and elements interact during an immune response. In addition, the model's parameters reflect the physiological features of the system, making the model appropriate for general use.

The remainder of this paper is organized as follows. First, the necessary biological background is presented. Next, related works are briefly discussed. This exposition is followed by a description of the mathematical model proposed in this work and the numerical scheme used to implement it. Then simulation results obtained from the proposed model are discussed, and, finally, our conclusions and plans for future work are presented.

## 2. Biological Background

The initial response of the host to a diverse array of biological stressors including bacterial infection, burns, trauma, and invasive surgery is an inflammatory response. Despite the growing understanding of the cellular and molecular mechanisms of inflammation, the complexity of the inflammatory response has challenged therapeutic development [3, 4]. A key reason for this conundrum has been speculated to be the difficulty of predicting the impact of manipulating individual components of the highly complex, nonlinear, and redundant inflammatory response [5]. Thus, progress would require a greater understanding of how components are organized. This makes systems biology based approaches appealing [6].

Most inflammatory reactions begin as a suppurative or purulent exudation process when the first line of cellular defense, the neutrophils, accumulate in the area. A suppurative process is characterized by the presence of pus (neutrophils mixed with cellular debris). Classically, there are three requisites of suppuration:

- presence of neutrophils that release proteolytic enzymes,
- necrosis of some types,
- liquefaction.

This suppurative process may lead to the formation of microabscesses. A microabscess is a localized collection of dead cells, body fluids, microbes, and other cells of the HIS. The process of formation of a microabscess begins when a cell of the HIS encounters bacteria and warn other cells that there is a stranger in the host. Its “warn” is in the form of a class of biochemicals called cytokines, which beckon other HIS cells to come to the point of infection and surround the enemy.

Most often all this goes unnoticed because the first few immune system cells phagocytize (engulf and digest) the invaders and the battle is finished. But every so often, an invader has a trick to escape the immune response and cannot be killed by the phagocytes. Those bacteria continue to grow and to spew out whatever they do. More and more immune system cells, mainly neutrophils, congregate at the infection site trapping the pathogens in the center. If this microabscess is close enough to the body surface, it can be seen as a blob of pus under the skin. When a microabscess is well developed, it has a wall or capsule of fibrous connective tissue separating it from the surrounding tissue, helping to prevent any microbes present in the microabscess from spreading to other areas of the body. Thus, microabscesses can be considered as a natural strategy used to fight against infection.

Microabscesses are found in many different diseases, for instance, the authers in [7–10] present animal studies detailing the formation of liver microabscess and microabscess by different types of infections. Epidermal microabscess formation by neutrophils was also evaluated in [11–14]. Infection of the heart by bacteria (bacterial myocarditis [15]) or by viruses (viral myocarditis [16]) is also correlated with microabscess formation by neutrophils. The interaction between tumor cells and inflammatory cells plays an important role in cancer initiation and progression and was investigated in [17] for the case of tumor-infiltrating neutrophils in pancreatic neoplasia, where the pattern of microabscess formation by neutrophils was reported once again.

## 3. Related Work

This section presents and discusses other mathematical and computational models of the immune response. Essentially, two distinct approaches are used: agent-based models and ordinary differential equations (ODEs). These models have some features in common with our model. All models include representative cells and molecules of the innate immune system. For example, neutrophils, macrophages, and proinflammatory cytokines are modeled in the majority of models. Some models as our model consider the important interactions between endothelial cells, tissue cells, and cytokines. Despite some similarities with our model, none of the works focus on modeling microabscesses.

## 4. Models Based on Agents

In [18, 19], it was developed an agent-based model of the dynamics of some cells, such as polymorphonuclear leukocytes (PMNs) and mononuclear cells and molecules, such as TNF-*α* and IL-1, during the initial inflammatory response in the interface endothelium/blood at the capillary level. Some characteristics of the model are as follows:

- all the cells are represented as agents whose behavior is close to the real;
- it considers the interactions between endothelial cells and circulating inflammatory cells at the blood/blood vessel-lining interface;
- the initial injury number (IIN) defines the number of tissue cells that are dead initially;
- the oxygen concentration is one important variable in the model;
- the total tissue damage is represented as a deficit in the oxygen variable;
- the injury state of an endothelial cell depends on the available oxygen concentration;
- proinflammatory mediators and endothelial cells surface adhesion molecules are modeled by state variables;
- it considers a generic pathogen that causes the infection.

The work aims to reproduce the time course of the early inflammatory response associated with the Systemic Inflammatory Syndrome Response (SIRS)/Multiple Organ Failure (MOF) from massive trauma or large exposure to endotoxin. The objective of the simulations were to compare the results with the soluble TNF-receptor experiment [20] where the soluble TNF-receptor is tested as a therapeutic treatment for the sepsis. The author claims that his results generally replicate the results of several large-scale clinical trials of cytokine-directed antimediator agents.

In [6], an agent-based modeling (ABM) framework is proposed to study the nonlinear dynamics of acute inflammatory responses to LPS. Their work uses an agent-based approach to elucidate molecular interactions involved in the NF-*κβ* signaling pathway, coupled with the spatial orientation of various inflammation specific molecules and cell populations such as macrophages and T-helper cells. In their model, the propagation of LPS signaling across the system is studied by considering the coupling between extracellular signals and transcriptional response. Some relevant characteristics considered in the model are as follows:

- molecular interactions,
- cellular heterogeneity,
- LPS/TLR4 signal transduction pathway,
- transcriptional response.

The proposed *in silico* model is evaluated through its ability to successfully reproduce a self-limited inflammatory response as well as a series of scenarios: a persistent (non)infectious response or innate immune tolerance and potentiation effects followed by perturbations in intracellular signaling molecules and cascades.

## 5. Models Based on ODEs

The model of [21] studies immunomodulatory strategies for treating cases of severe sepsis. They introduced and evaluated the concept of conducting a randomized clinical trial *in silico* based on simulated patients generated from a mechanistic mathematical model of bacterial infection, acute inflammatory response, global tissue dysfunction, and a therapeutic intervention. Trial populations are constructed to reflect heterogeneity in bacterial load and virulence as well as propensity to mount and modulate an inflammatory response. They constructed a cohort of 1000 trial patients submitted to therapy with one of three different doses of a neutralizing antibody directed against tumor necrosis factor (anti-TNF) for 6, 24, or 48 hrs. Their focus was to assess the feasibility of using differential equation models to improve the design of clinical trials. This paper replicates *in silico* the general findings from actual clinical trials—that it is very difficult to design a treatment strategy that is effective over a broad range of sepsis patients.

## 6. Hybrid Models

In [22], a hybrid model that coupled an agent-based model [18, 19] and a system dynamics/differential equation model [21] was created using the System Dynamics tool within Netlogo [23]. They developed an interface between the agent-based and system dynamics models. The area of initial infection and subsequent interactions at this point were simulated with the agent-based model and certain aspects primarily related to the production and life cycle of circulating inflammatory cells was modeled with the system dynamics model. They implemented a simple system dynamics model that focused on systemic polymorphoneutrophils (PMN) production, maturation, sequestration, and release. Some of the flows inside the system dynamics model were influenced by the conditions within the agent-based model. The primary role of the system dynamics model was to manifest a delay between the elevation of the cytokines in the tissue and the increase in PMNs in the circulating blood. The objective of the work was to reproduce some of the results of the agent-based model [18, 19] using the hybrid model.

## 7. Mathematical Model

Our main objective is to develop a parameterized mathematical model of the human innate immune system that simulates the immune response occurring in a generic tissue. To achieve this goal, we first build a model of the immune response to LPS [1, 2]. In this work, we extend this model to reproduce the spatiotemporal dynamics of a bacterial infection and the process of microabscess formation.

The mathematical model simulates the temporal and spatial behavior of bacteria (*B*), dead bacteria (BD), macrophages, neutrophils (*N*), apoptotic neutrophils (ND), proinflammatory cytokines (CH), healthy tissue cells (HT), and dead tissue cells (TD). Macrophages are present in two states of readiness: resting (RM) and *hyperactivated* (AM). We must stress that the equations modeling proinflammatory cytokines are generic in the sense that they model the role of distinct cytokines taking part in the inflammatory process. Equation parameters can be adjusted to model the role of a specific proinflammatory cytokine.

The relationships among all of the model's components are described next. Neutrophils, resting macrophages, and active macrophages phagocytose the bacteria. The neutrophils then undergo apoptosis, which may or may not be induced by the phagocytosis process. In this different state, apoptotic neutrophils cannot perform phagocytosis or produce proinflammatory cytokines; as a result, apoptotic neutrophils are eliminated from the body after being phagocytosed by active macrophages. Apoptotic neutrophils will die after a period of time, releasing cytotoxic granules and degradation enzymes in the medium that cause tissue damage destroying healthy tissue cells. Active neutrophils and bacteria also cause tissue damage by producing toxic products that are not modeled here. The infection site is “cleaned” by resting and active macrophages that do the phagocytosis of dead tissue cells. Healthy tissue cells in contact with bacteria, neutrophils, and active macrophages produce proinflammatory cytokines. The proinflammatory cytokines increase the permeability of the blood vessels; consequently, more neutrophils and monocytes are recruited to the infected tissue. In addition, the proinflammatory cytokines act as a chemoattractant substance to the resting macrophages, active macrophages, and neutrophils.

Below, we provide the equations derived from the model. Equation (1) provides the bacteria differential equation:

(1) ∂B∂t=rB·B·g(w)−μB·B−λN|B·N·B−λRM|B·RM·B−λAM|B·AM·B+DB·dif(B,w),B(x,y,0)=B0,  ∂B(·,t)∂n|∂Ω=0.

In this equation, *r*_*B*_ · *B* · *g*(*w*) denotes the reproduction term of the bacteria, where *r*_*B*_ is the rate of reproduction and *g*(*w*) is a function of the total density of cells *w* in a discretized area of the two-dimensional space at a specific time step. The *w* variable is defined as

(2) w(x,y,t)=B(x,y,t)+BD(x,y,t)+N(x,y,t)+ND(x,y,t)+RM(x,y,t)+AM(x,y,t)+CH(x,y,t)+HT(x,y,t)+TD(x,y,t);

*μ*_*B*_ · *B* denotes the decay of bacteria, where *μ*_*B*_ is the rate of decay. *λ*_*N*|*B*_ · *N* · *B* denotes the phagocytosis of bacteria by neutrophils, where *λ*_*N*|*B*_ is the rate of this phagocytosis. *λ*_RM|*B*_ · RM · *B* denotes the phagocytosis of bacteria by resting macrophages, where *λ*_RM|*B*_ is the rate of this phagocytosis. *λ*_AM|*B*_ · AM · *B* denotes the phagocytosis of bacteria by active macrophages, where *λ*_AM|*B*_ is the rate of this phagocytosis. *D*_*B*_ · dif(*B*, *w*) denotes bacteria diffusion, where *D*_*B*_ represents the diffusion coefficient and dif(*B*, *w*) is calculated in the following way:

(3) $$\begin{array}{c} \text{dif}\left(B,w\right)=\nabla \cdot \left(g\left(w\right)\nabla \left(f\left(w\right)B\right)-f\left(w\right)B\nabla g\left(w\right)\right). \end{array}$$

The *f* function models the probability of a cell being pushed from a site due to the pressure exerted by neighboring cells [24, 25]. This population pressure is modelled by a Hill equation [26]. It increases with the total density of cells *w* occupying the same position in space and has a saturation in a high density of cells.

The *f* function is defined as

(4) $$\begin{array}{c} f\left(w\right)=1+\alpha \frac{w}{\beta +w}. \end{array}$$

*α* and *β* are constant values.

The *g* function returns the percentage of free space in a discretized area of the two-dimensional space and its use is motivated by some important biological concepts such as quorum sensing/volume sensing [24, 25, 27–29]. The idea is that cells have a set of cell density sensing mechanisms and changes its behavior in crowded regions. In the context of our model it is used to limit the density of cells that occupy a discretized area of our two-dimensional domain. The *g* function is defined as

(5) $$\begin{array}{c} g\left(w\right)=1-\frac{w}{\text{total}}. \end{array}$$

The variable total represents the maximum density of cells that fits in a discretized area of the tissue.

The differential equation corresponding to dead bacteria (BD) is given as follows:

(6) ∂BD∂t=μB·B+λN|B·N·B+λRM|B·RM·B+λAM|B·AM·B−λAM|BD·AM·BD−λRM|BD·RM·BD+DBD·dif(BD,w),BD(x,y,0)=B0,  ∂BD(·,t)∂n|∂Ω=0.

Here, note that *μ*_*B*_ · *B*, *λ*_*N*|*B*_ · *N* · *B*, *λ*_RM|*B*_ · RM · *B* and *λ*_AM|*B*_ · AM · *B* were defined previously. *λ*_AM|BD_ · AM · BD denotes the phagocytosis of dead bacteria by active macrophages, where *λ*_AM|BD_ is the rate of phagocytosis. *λ*_RM|BD_ · RM · BD denotes the phagocytosis of dead bacteria by resting macrophages, where *λ*_RM|BD_ is the rate of phagocytosis. *D*_BD_ · dif(BD, *w*) denotes dead bacteria diffusion, where *D*_BD_ represents the diffusion coefficient and the function dif was defined previously.

The differential equation corresponding to the resting macrophage (RM) is given as follows:

(7) PRM=(PRMmax⁡−PRMmin⁡)·CH(CH+keqCH)+PRMmin⁡,sourceRM=PRM·(Mmax⁡−(RM+AM)),∂RM∂t=−μRM·RM−λB|RM·B·RM+sourceRM·g(w)+DRM·dif(RM,w)−χRM·chemotaxis(RM,CH,w),RM(x,y,0)=RM0,  ∂RM(·,t)∂n|∂Ω=0.

*P*_RM_ denote the increase in endothelium permeability and its effects on monocyte extravasation. The permeability of blood vessel endothelium is modeled by a Hill equation [26], which also has been used to model drug dose-response relationships [30]. The idea is to model the increase in the permeability of the endothelium in accordance with the number of proinflammatory cytokines deposited on the endothelium.

The calculation of *P*_RM_ involves the following parameters: (a) *P*_RM_^max⁡^, the maximum endothelium permeability induced by the proinflammatory cytokine; (b) *P*_RM_^min⁡^, the minimum endothelium permeability induced by the proinflammatory cytokine; (c) keq_CH_, the number of proinflammatory cytokines that exert 50% of the maximum effect on permeability. source_RM_ · *g*(*w*) denotes the source term of macrophages, which is related to the number of monocytes that will enter into the tissue from the blood vessels. This number depends on the endothelium permeability *P*_RM_ and on the number of monocytes appearing in the blood (*M* ^max⁡^).

*μ*_RM_RM denotes resting macrophage apoptosis, where *μ*_RM_ is the apoptosis rate. *λ*_*B*|RM_ · *B* · RM denotes the activation of resting macrophages, where *λ*_*B*|RM_ is the rate of activation. *D*_RM_ · dif(RM, *w*) denotes resting macrophage diffusion, where *D*_RM_ represents the diffusion coefficient and the function dif was defined previously. *χ*_RM_ · chemotaxis(RM, CH, *w*) denotes resting macrophage chemotaxis, where *χ*_RM_ is the chemotaxis rate and chemotaxis(RM, CH, *w*) is calculated in the following way:

(8) $$\begin{array}{c} \text{chemotaxis}\left(\text{RM},\text{CH},w\right)=\nabla \cdot \left(\text{RM}g\left(w\right)f\left(w\right)\nabla \text{CH}\right). \end{array}$$

The differential equation corresponding to the active macrophage (AM) is given as follows:

(9) ∂AM∂t=−μAM·AM+λB|RM·B·RM+DAM·dif(AM,w)−χAM·chemotaxis(AM,CH,w),AM(x,y,0)=AM0,  ∂AM(·,t)∂n|∂Ω=0.

Here, note that *λ*_*B*|RM_ · *B* · RM was defined previously. Above, *μ*_AM_ · AM, *D*_AM_ · dif(AM, *w*), and *χ*_AM_ · chemotaxis(AM, CH, *w*) denote the active macrophage apoptosis, diffusion, and chemotaxis, respectively, whereas *μ*_AM_, *D*_AM_, and *χ*_AM_ are the apoptosis rate, diffusion coefficient, and chemotaxis rate, respectively.

The neutrophil differential equation (*N*) is given as follows:

(10) PN=(PNmax⁡−PNmin⁡)·CHCH+keqCH+PNmin⁡,sourceN=PN·(Nmax⁡−N),∂N∂t=−μN·N−λB|N·B·N+sourceN·g(w)+DN·dif(N,w)−χN·chemotaxis(N,CH,w),N(x,y,0)=N0,  ∂N(·,t)∂n|∂Ω=0.

In this equation, *P*_*N*_ denotes the increase in endothelium permeability and its effects on neutrophil extravasation. In the top equation, *P*_*N*_^max⁡^ is the maximum endothelium permeability induced by proinflammatory cytokines, *P*_*N*_^min⁡^ is the minimum endothelium permeability induced by proinflammatory cytokines, and keq_CH_ is the number of proinflammatory cytokines that exert 50% of the maximum effect on endothelium permeability.

Here, *μ*_*N*_ · *N* denotes neutrophil apoptosis, where *μ*_*N*_ is the rate of apoptosis. *λ*_*B*|*N*_ · *B* · *N* denotes the neutrophil apoptosis induced by phagocytosis, where *λ*_*B*|*N*_ represents the rate of this induced apoptosis. source_*N*_ · *g*(*w*) represents the source term of neutrophil, that is, the number of neutrophils entering the tissue from the blood vessels. This number depends on the endothelium permeability (*P*_*N*_) and on the number of neutrophils in the blood (*N* ^max⁡^).

*D*_*N*_ · dif(*N*, *w*) denotes neutrophil diffusion, where *D*_*N*_ represents the diffusion coefficient and the function dif was defined previously. *χ*_*N*_ · chemotaxis(*N*, CH, *w*) denotes neutrophil chemotaxis, where *χ*_*N*_ is the chemotaxis rate and chemotaxis(*N*, CH, *w*) was defined previously.

The differential equation corresponding to the apoptotic neutrophil (ND) is given as follows:

(11) ∂ND∂t=μN·N+λB|N·B·N−λND|AM·ND·AM−μNDND+DND·dif(ND,w),ND(x,y,0)=ND0,  ∂ND(·,t)∂n|∂Ω=0.

Here, note that *μ*_*N*_ · *N* and *λ*_*B*|*N*_ · *B* · *N* were defined previously, whereas *λ*_ND|AM_ · ND · AM denotes the phagocytosis of the apoptotic neutrophil carried out by active macrophages, and *λ*_ND|AM_ is the rate of this phagocytosis. *μ*_ND_ND denotes the neutrophil necrosis, where *μ*_ND_ is the rate of necrosis. *D*_ND_ · dif(ND, *w*) denotes apoptotic neutrophil diffusion, where *D*_ND_ represents the diffusion coefficient and the function dif was defined previously.

The differential equation for the proinflammatory cytokine (CH) is given in as follows:

(12) ∂CH∂t=−μCH·CH+(βCH|N·N·B+βCH|AM·AM·B+βCH|HT·HT·B)·g(w)+DCH·dif(CH,w)CH(x,y,0)=CH0,  ∂CH(·,t)∂n|∂Ω=0.

In this equation, *μ*_CH_CH denotes the proinflammatory cytokine decay, where *μ*_CH_ is the decay rate. *β*_CH|*N*_ · *N* · *B* denotes the proinflammatory cytokine production by the neutrophils, where *β*_CH|*N*_ is the production rate. *β*_CH|AM_ · AM · *B* denotes the proinflammatory cytokine production by active macrophages, where *β*_CH|AM_ is the production rate. *β*_CH|HT_ · HT · *B* denotes the proinflammatory cytokine production by healthy tissue cells in contact with bacteria, where *β*_CH|HT_ is the production rate. *D*_CH_ · dif(CH, *w*) denotes the proinflammatory cytokine diffusion, where *D*_CH_ represents the diffusion coefficient and the function dif was defined previously.

The differential equation corresponding to the healthy tissue (HT) is given as follows:

(13) $$\begin{array}{c} \frac{\partial \text{HT}}{\partial t}=-\mu_{\text{ND}}\text{ND}-\lambda_{B|\text{HT}}\cdot B\cdot \text{HT},\\ \text{HT}\left(x,y,0\right)={\text{HT}}_{0},\;\;{\frac{\partial \text{HT}\left(\cdot ,t\right)}{\partial n}|}_{\partial \Omega }=0. \end{array}$$

*μ*_ND_ND denotes the tissue damage caused by the release of toxic products from necrotic neutrophils. *λ*_*B*|HT_ · *B* · HT denotes the tissue damage caused by bacteria, where *λ*_*B*|HT_ is the rate of damage.

The differential equation corresponding to the dead tissue (TD) is given as follows:

(14) ∂TD∂t=μNDND+λB|HT·B·HT−λRM|TD·RM·TD−λAM|TD·AM·TD,TD(x,y,0)=TD0,  ∂TD(·,t)∂n|∂Ω=0.

*μ*_ND_ND and *λ*_*B*|HT_ · *B* · HT were defined previously. *λ*_RM|TD_ · RM · TD denotes the phagocytosis of dead tissue cells by resting macrophages, where *λ*_RM|TD_ is the rate of phagocytosis. *λ*_AM|TD_ · AM · TD denotes the phagocytosis of dead tissue cells by active macrophages, where *λ*_AM|TD_ is the rate of phagocytosis.

The mathematical model presented here introduced some modifications to our previous model [2] with the aim to reproduce the microabscess formation. We included equations for the dynamics of the tissue to take into account some effects of infection such as tissue damage and production of cytokines by tissue cells. We also replaced the LPS equation in our previous model [2] by the bacteria equation with a term for reproduction of bacteria. Besides we modified the calculus of the diffusion and chemotaxis terms [24] (a) to limit the number of cells that are allowed to stay at the same time in the same area of the domain and (b) to reduce the efficiency of the diffusion and chemotaxis processes in overcrowded regions. More specifically, the method we implemented incorporates the following general mechanisms which may lead to dispersal of the population [24].

- Population pressure: we assume that a high cell density results in increased probability of a cell being pushed from a site, for example, due to the pressure exerted by neighboring cells. This is achieved phenomenologically with the *f* function of our mathematical model and the changes in diffusion and chemotaxis calculus.
- Limited space: here we assume that no more cells can enter a site above a total cell density. In our model, this is achieved with the incorporation of the *g* function in the diffusion and chemotaxis calculations.
- Gradient detection: cells may detect and respond to a local gradient in the cell density and as a consequence cells can move to higher concentrations of the attractant substance.

## 8. Implementation

 The numerical method used to solve the mathematical model was the Finite Difference Method [31], a method commonly used in the numeric discretization of PDEs.

A complex part of the resolution of the PDEs is the resolution of the convective term, the chemotaxis term. The development of numerical methods to approximate convective terms (in most cases not linear) have been subject of intense researches [32–35].

Different numerical approaches have been proposed for the discretization of the chemotaxis term [36, 37]. Our implementation is based on the finite difference method for the spatial discretization and the explicit Euler method for the time evolution. The discretization of the chemotaxis term (∇·(*χ*_*N*_ *N*∇CH)) uses the First-Order Upwind scheme [38]. Therefore, the precision of our numerical implementation is first-order in time (explicit Euler) and first-order in space (upwind scheme). The upwind scheme discretizes the hyperbolic PDEs through the use of differences with bias in the direction given by the signal of the characteristics' speeds. The upwind scheme uses an adaptive or solution-sensitive stencil to numerically simulate more precisely the direction of information propagation.

In two-dimension, the upwind scheme approximates the chemotaxis term as the sum of the flux in the *x* direction (res*X*) with the flux in the *y* direction (res*Y*). res*X* is the sum of the flux_left at the point *x* − delta*X*/2 with the flux_right at the point *x* + delta*X*/2 and res*Y* is the sum of the flux_up at the point *y* − delta*Y*/2 with the flux_down at the point *y* + delta*Y*/2 in Algorithm 1.

In this code, ch represents the discretization of the proinflammatory cytokine, *n* represents the discretization of neutrophils, *w* is the total density of cells in a position of the space, *x* and *y* are the positions in space, and delta*X* and delta*Y* are the spatial discretizations in *x* and *y* directions, respectively. The test made is to define what is the signal of the characteristic speed, where the speed of the movement of *N*(*y*, *x*) is given by the term ∇CH. This value is then used to choose between two schemes of finite differences: forward or backward.

We decided to implement our own numerical method to solve the systems of PDEs because (a) we have the possibility to parallelize the code and (b) most of the numerical libraries offer few functions that are suitable to our problem. The sequential code was implemented in C.

## 9. Numerical Experiments

We performed several simulations in order to verify that the model's results are in agreement with what is described in the literature. Our objective was to reproduce some characteristics of the microabscess such as an accumulation of dead cells and bacteria in the infection site.

The model's initial conditions and parameters are given in Tables 1 and 2, respectively.

In Table 2, parameters marked with * were adjusted to qualitatively reproduce the results obtained in several studies of the immune response to LPS. In the case of the bacteria (results not shown here), we adjust the equation parameters in order to obtain an exponential decrease in time as shown in [39]. The results of the concentration of proinflammatory cytokines in time (results not shown here) are qualitatively similar to those obtained in some experimental works [40–42]. The parameters marked with ** were based on the values given in the references but were adjusted due to the use of distinct units (e.g., from liter to mm^3^) or to fit in a 25 mm^2^ tissue. In this paper, we obtained parameter values for humans whenever they were available. The variables *β* and total in (5) and (4) were defined based on the concentrations of neutrophils and macrophages per liter given in [43] and were adjusted (a) due to the use of distinct units (e.g., from liter to mm^3^) and (b) to fit in a 25 mm^2^ tissue. The variable total represents the maximum density of cells that fits in a discretized area of the tissue.

In the next sections, we will show the results of the simulation performed with the parameters given in Table 2. In this simulation, we considered a 5 mm × 5 mm two-dimensional domain representing a tissue with 25 mm^2^ of area and a simulation time of 5 days. In our model, the exchange between the vascular system (arterioles and vessels) and tissue was assumed to occur only at the points (1,1), (1,4), and (4,2.5), In this point, immune cells (neutrophils and macrophages) that are in the blood stream can enter into the tissue. The communication between blood vessels and tissue is modeled by permeabilities that vary in time and may depend on the concentration of different cells and molecules (in our model, the endothelium permeability of neutrophils and macrophages depends on the concentration of the proinflammatory cytokine).

## 10. Bacteria

**Figure 1:**
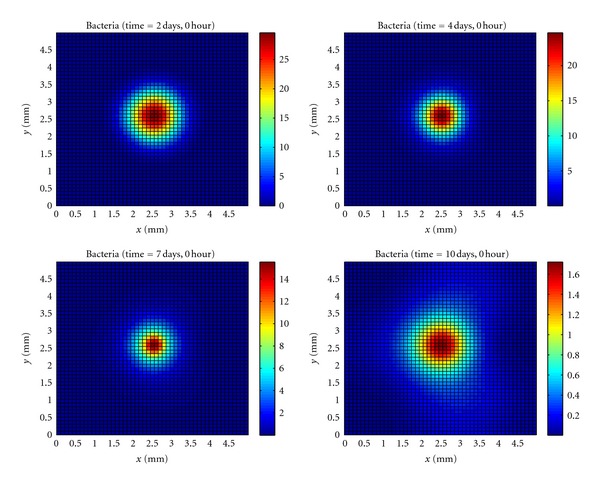
Temporal evolution and spatial distribution of bacteria.

In the case of bacteria (Figure 1), we observe that initially the bacteria diffuses through the tissue causing tissue damage without its presence to be noticed.

As soon as resting macrophages residents in the tissue recognize the bacteria they start to produce proinflammatory cytokines that will diffuse through the tissue reaching the blood vessel. Once proinflammatory cytokines interact with the endothelial cells an increase in the endothelium permeability occurs allowing neutrophils and monocytes to migrate to the tissue.

The bacteria starts to die a lot due to the presence of huge numbers of neutrophils. However, the immune response can not completely eliminate bacteria due to the formation of the microabscess pattern. In the microabscess, there are bacteria and a huge concentration of dead cells around it (Figures 4, 5, and 6). In this context, the cleaning process realized by macrophages is very important to allow neutrophils to reach bacteria and eliminate them. Macrophages are responsible for phagocyte dead cells that accumulated in the microabscess. The pattern of microabscess could have lasted longer if we had considered the formation of fibrous tissue around the microabscess.

## 11. Neutrophil

**Figure 2:**
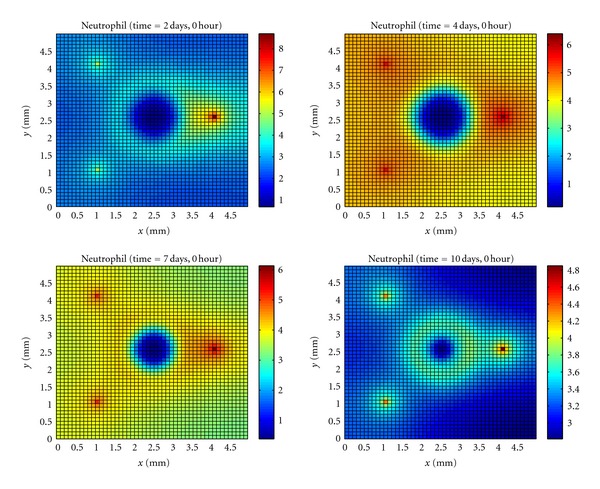
Temporal evolution and spatial distribution of neutrophil.

Neutrophils are initially attracted to the tissue by proinflammatory cytokines produced by activated resident macrophages (Figure 2). Once a neutrophil encounters bacteria, it phagocytizes bacteria and starts to produce proinflammatory cytokines that will attract more neutrophils and macrophages. The cytokine gradient will guide the movement of neutrophils and macrophages in the direction of the highest bacteria concentration.

After the microabscess formation, the immune system cells lose contact with a high number of bacteria. As these cells tend to move following the cytokine gradient, we can observe an accumulation of them around the microabscess.

After a significant number of macrophages phagocyte dead cells, the neutrophils can encounter the bacteria and phagocyte them from the border to the center of the microabscess area. As a consequence, a reduction in the microabscess area is observed (Figure 1), which indicates that the immune response is succeeding in controlling the infection.

## 12. Cytokine

**Figure 3:**
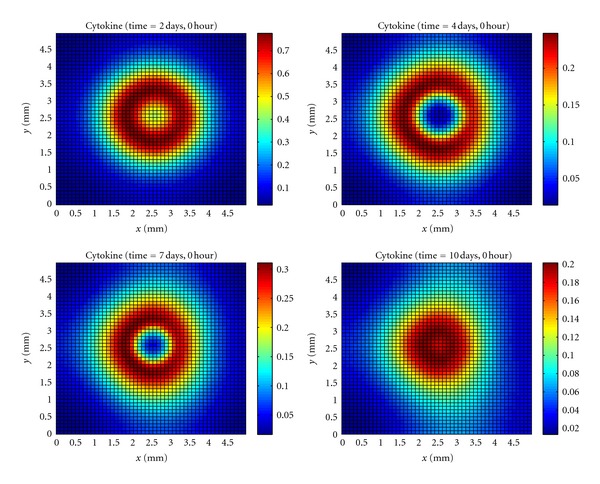
Temporal evolution and spatial distribution of cytokine.

The cytokines in Figure 3 are produced primarily by resident macrophages that are the first to recognize the bacteria presence. The cytokines will increase the endothelium permeability allowing neutrophils to migrate to the tissue. The arrived neutrophils will produce even more cytokines that will guide the movement of neutrophils and macrophage cells in the direction of high concentrations of bacteria.

During the formation of the microabscess and after it, the production of cytokines is higher in the regions where neutrophils and macrophages have contact with the bacteria that is surrounding the microabscess.

## 13. Apoptotic Neutrophils

**Figure 4:**
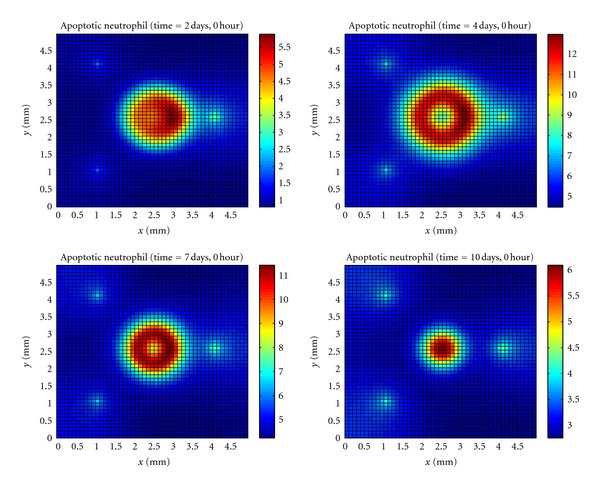
Temporal evolution and spatial distribution of apoptotic neutrophil.

In Figure 4, it can be observed that initially the neutrophils that came from the blood vessel closer to the site of infection died in large number than the neutrophils that came from other sites. Then, after microabscess formation, a lot of neutrophils start to die around the entire microabscess. This phenomenon continues until the microabscess disappears.

## 14. Dead Bacteria

**Figure 5:**
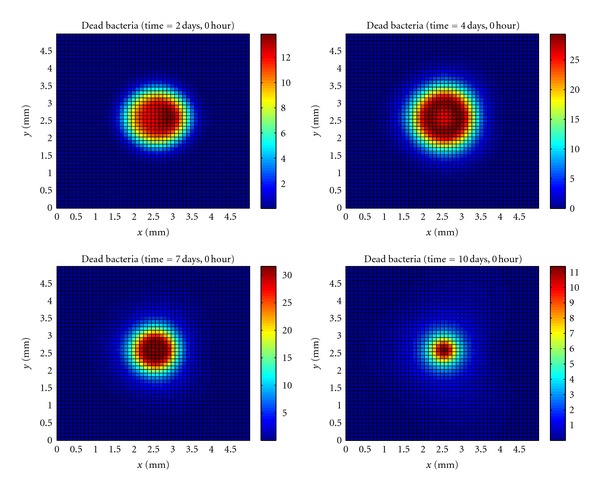
Temporal evolution and spatial distribution of dead bacteria.

Figure 5 shows that initially more bacteria died near blood vessels. After the formation of the microabscess, the bacteria starts to die around the entire microabscess since this is the area where the immune response is acting.

## 15. Tissue Cells

**Figure 6:**
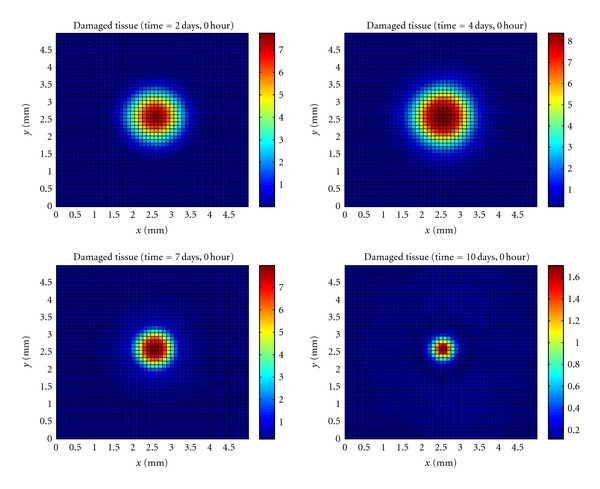
Temporal evolution and spatial distribution of damaged tissue cells.

In Figure 6, it can be observed that a lot of tissue damage by bacteria during the period the immune system took to mount an effective immune response. The number of dead tissue cells then reduces, because of the phagocytosis realized by macrophages.

**Figure 7:**
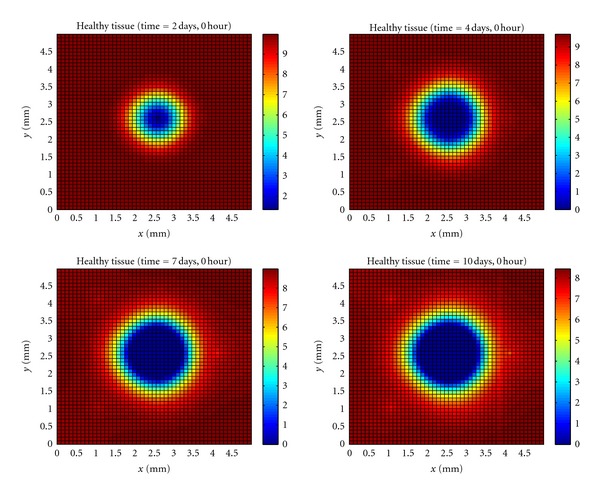
Temporal evolution and spatial distribution of healthy tissue cells.

Figure 7 shows the evolution of the healthy tissue area destroyed by the bacteria.

## 16. Microabscess Area

**Figure 8:**
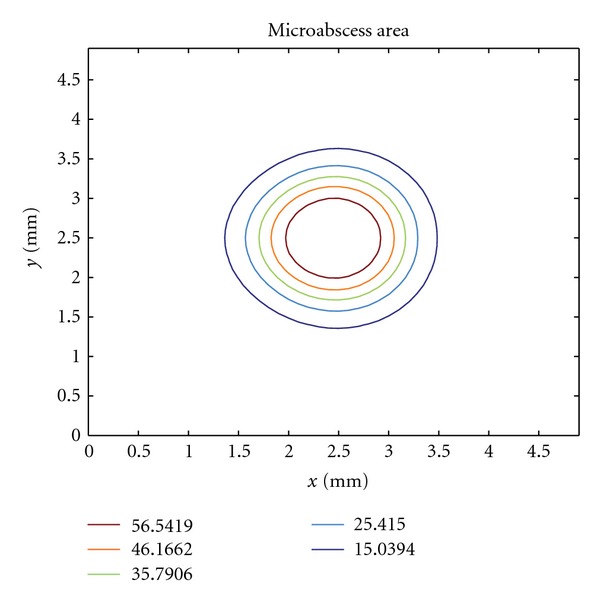
Level curves highlighting the microabscess area at day 5 of the immune response.

Figure 8 shows the microabscess area using a set of level curves. We defined the microabscess as an area where the concentration of bacteria plus concentration of dead bacteria plus the concentrations of damaged tissue and apoptotic neutrophil is higher. These results show that our model was capable to reproduce the formation of the microabscess in agreement with the observed characteristics of a microabscess [7, 14, 49–54].

## 17. Conclusions and Future Works

In this work, we presented a computational model for the dynamics of representative types of cells and molecules of the HIS during an immune response to a bacteria. Despite the simplifications and limitations of the model, our results showed that we were able to reproduce an initial microabscess formation. The spatial results show a collection of dead tissue cells, dead bacteria, and apoptotic neutrophil in the microabscess region. This distinct pattern of formation can only be reproduced by spatiotemporal models, such as PDEs models.

As future work, we plan to perform a detailed sensitivity analysis of our microabscess formation model. A previous work [55] has given us some hints about the most sensitivity parameter of the model. We also plan to validate our model against experimental data.

We also plan to modify many aspects of the model to make it more real. For example, we plan to consider a more adequate model to represent the structure of the tissue and its constituents. The tissue can be better characterized as a multiphasic porous medium subjected to stress and deformation variations mainly during the inflammatory process. This porous medium would comprise fluids, extracellular matrix, cells, and molecules. We also plan to model the mechanical behaviour of each of these phases and the mechanical interactions between them.

We have interest in developing models for processes such as vasodilation, coagulation, and others and analyse its effects on the mechanical behaviour of immune system cells and the consequences for the immune response.

With the aim to investigate better the formation of the microabscess, we plan to add another features that contribute to this formation such as the effects of extracellular pH on immune response [56]. Acidic pH predominates at inflammatory *loci* and other sites of immune activity. Investigations on neutrophils demonstrate mainly inhibition of chemotaxis, respiratory activity, and bactericidal capacity at reduced pH. Besides diminished extracellular pH may play a role in suppressing cytokine production and cytotoxic activities by pulmonary macrophages [56].

Besides we plan to add to the model the process of fibrous tissue formation around the microabscess. We plan to investigate what factors determine if the fibrous tissue will be produced or not. The production of fibrous tissue as well as the coagulation process are ways of the immune system to prevent the bacteria to spread throughout the body doing damage with possible serious consequences, for example, SIRS/MOF. In particular, we are interested in modeling the participation of macrophages, fibroblasts, tissue cells, endothelial cells, and many mediators in the process of fibrous tissue formation. An important step in this process is the production of collagen by fibroblasts induced by the cytokine TGF-*β* produced by macrophages [57, 58]. The macrophages has many roles in the processes of wound healing and tissue repair. For example, during the coagulation process, macrophages and endothelial cells are responsible for the production of diverse growth factors and chemotcatic substances that attracts and stimulates the proliferation of tissue cells initiating tissue repair [59].

## Figures and Tables

**Algorithm 1:**
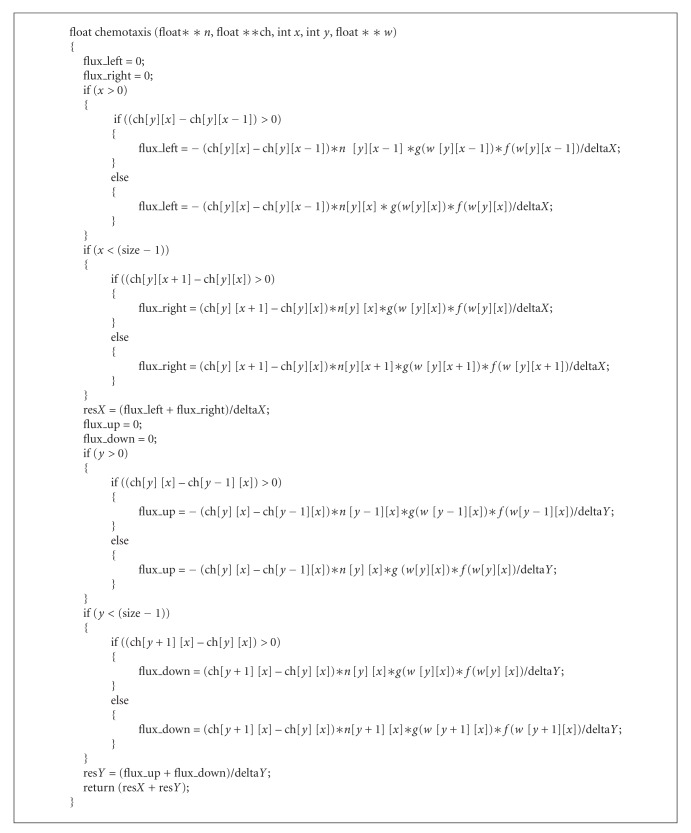


**Table 1:** Initial conditions.

Parameter	Value	Unit
B_0_	$\{\begin{array}{c} 70:\;x=2.5\;\text{mm},\;\;y=2.5\;\text{mm}\\ 0:\;\text{otherwise} \end{array}$	10^4^ cells/mm^3^
BD_0_	$\begin{array}{c} 0:\;0\leq x\leq 4,0\leq y\leq 4\;\; \end{array}$	10^4^ cells/mm^3^
RM_0_	$\{\begin{array}{c} 10:\;x=1\;\text{mm},\;\;y=1\;\text{mm}\\ 10:\;x=1\;\text{mm},\;\;y=4\;\text{mm}\\ 10:\;x=4\;\text{mm},\;\;y=2.5\;\text{mm}\\ 0:\;\;\text{otherwise} \end{array}$	10^4^ cells/mm^3^
AM_0_	$\begin{array}{c} 0:\;0\leq x\leq 4,0\leq y\leq 4\;\;\; \end{array}$	10^4^ cells/mm^3^
N_0_	0 : 0 ≤ *x* ≤ 4,0 ≤ *y* ≤ 4	10^4^ cells/mm^3^
ND_0_	0 : 0 ≤ *x* ≤ 4,0 ≤ *y* ≤ 4	10^4^ cells/mm^3^
CH_0_	$\begin{array}{c} 0:\;0\leq x\leq 4,0\leq y\leq 4\;\; \end{array}$	10^4^ cells/mm^3^
HT_0_	10 : 0 ≤ *x* ≤ 4,0 ≤ *y* ≤ 4	cells/mm^3^
TD_0_	0 : 0 ≤ *x* ≤ 4,0 ≤ *y* ≤ 4	10^4^ cells/mm^3^

**Table 2:** Parameters.

Parameter	Value	Unit	Reference
α	0.05	Adimensional	Estimated*
β	35	Cells/mm^3^	Estimated*
Total	70	Cells/mm^3^	Estimated*
*M* ^max⁡^	15000	Cells/mm^3^	[44]**
*N* ^max⁡^	250000	Cells/mm^3^	[44]**
*P*_*N*_^max⁡^	1	1/day	[45]**
*P*_RM_^max⁡^	1	1/day	Estimated*
*P*_*N*_^min⁡^	0.001	1/day	Estimated*
*P*_RM_^min⁡^	0.01	1/day	Estimated*
keq_CH_	5	Cells/mm^3^	Estimated*
*r*_*B*_	4	1/day	[46]
λ_*B*∣HT_	0.05	1/(cells/mm^3^)·day	[47]
λ_AM∣BD_	0.6	1/(cells/mm^3^)·day	[47]**
λ_AM∣ND_	0.8	1/(cells/mm^3^)·day	[44]
λ_AM∣TD_	0.6	1/(cells/mm^3^)·day	[47]**
λ_RM∣BD_	0.6	1/(cells/mm^3^)·day	[47]**
λ_RM∣TD_	0.6	1/(cells/mm^3^)·day	[47]**
λ_N∣B_	0.55	1/(cells/mm^3^)·day	[44]
λ_B∣N_	0.24	1/(cells/mm^3^)·day	[47]
λ_RM∣*B*_	0.25	1/(cells/mm^3^)·day	[44]
λ_AM∣*B*_	0.8	1/(cells/mm^3^)·day	[44]
μ_*B*_	0.01	1/day	[44]
μ_*N*_	0.67	1/day	[44]
μ_ND_	0.05	1/day	[44]
μ_RM_	0.0033	1/day	[44]
μ_AM_	0.07	1/day	[44]
μ_CH_	12	1/day	[44]
*D*_*B*_	0.05	mm^2^/day	[44]
*D*_RM_	5	mm^2^/day	[44]
*D*_AM_	5	mm^2^/day	[44]
*D*_*N*_	10	mm^2^/day	[44]
*D*_ND_	0.001	mm^2^/day	[44]
*D*_CH_	6	mm^2^/day	[44]
χ_*N*_	10	mm^2^/day	[44]
χ_RM_	5	mm^2^/day	[44]
χ_AM_	7	mm^2^/day	[44]
β_CH∣*N*_	1	1/(cells/mm^3^)·day	[48]*
β_CH∣AM_	1	1/(cells/mm^3^)·day	[48]*
β_CH∣HT_	0.2	1/(cells/mm^3^)·day	[48]*
RM_act_	0.4	1/(cells/mm^3^)·day	[44]

## References

[1] Pigozzo AB, Macedo GC, Dos Santos RW, Lobosco M Implementation of a computational model of the innate immune system.

[2] Pigozzo AB, Macedo GC, dos Santos RW, Lobosco M On the computational modelling of the innate immune system.

[3] Kerschen EJ, Fernandez JA, Cooley BC (2007). Endotoxemia and sepsis mortality reduction by non-anticoagulant-activated protein C. *Journal of Experimental Medicine*.

[4] Klaitman VK, Almog Y (2003). Corticosteroids in sepsis: a new concept for an old drug. *Israel Medical Association Journal*.

[5] Vodovotz Y, Constantine G, Rubin J, Csete M, Voit EO, An G (2009). Mechanistic simulations of inflammation: current state and future prospects. *Mathematical Biosciences*.

[6] Dong X, Foteinou PT, Calvano SE, Lowry SF, Androulakis IP (2010). Agent-based modeling of endotoxin-induced acute inflammatory response in human blood leukocytes. *PLoS ONE*.

[7] Seydel KB, Zhang T, Stanley SL (1997). Neutrophils play a critical role in early resistance to amebic liver abscesses in severe combined immunodeficient mice. *Infection and Immunity*.

[8] Rigothier MC, Khun H, Tavares P, Cardona A, Huerre M, Guillén N (2002). Fate of Entamoeba histolytica during establishment of amoebic liver abscess analyzed by quantitative radioimaging and histology. *Infection and Immunity*.

[9] Lin JC, Chang FY, Fung CP (2010). Do neutrophils play a role in establishing liver abscesses and distant metastases caused by Klebsiella pneumoniae?. *PLoS ONE*.

[10] Ebe Y, Hasegawa G, Takatsuka H (1999). The role of Kupffer cells and regulation of neutrophil migration into the liver by macrophage inflammatory protein-2 in primary listeriosis in mice. *Pathology International*.

[11] Schön M, Denzer D, Kubitza RC, Ruzicka T, Schön MP (2000). Critical role of neutrophils for the generation of psoriasiform skin lesions in flaky skin mice. *Journal of Investigative Dermatology*.

[12] Wetzel A, Wetzig T, Haustein UF (2006). Increased neutrophil adherence in psoriasis: role of the human endothelial cell receptor Thy-1 (CD90). *Journal of Investigative Dermatology*.

[13] Grayson W (2008). The HIV-positive skin biopsy. *Journal of Clinical Pathology*.

[14] Cybulsky MI, Cybulsky IJ, Movat HZ (1986). Neutropenic responses to intradermal injections of *Escherichia coli.* effects on the kinetics of polymorphonuclear leukocyte emigration. *American Journal of Pathology*.

[15] Bhavsar J, Gordon D, Shea M (2010). Listeria myocarditis with an atypical intracavitary vegetation/thrombus. *Heart*.

[16] Oka K, Oohira K, Yatabe Y (2005). Fulminant myocarditis demonstrating uncommon morphology—a report of two autopsy cases. *Virchows Archiv*.

[17] Reid MD, Basturk O, Thirabanjasak D (2011). Tumor-infiltrating neutrophils in pancreatic neoplasia. *Modern Pathology*.

[18] An G (2001). Agent-based computer simulation and sirs: building a bridge between basic science and clinical trials. *Shock*.

[19] An G (2004). In silico experiments of existing and hypothetical cytokine-directed clinical trials using agent-based modeling. *Critical Care Medicine*.

[20] Fisher CJ, Agosti JM, Opal SM (1996). Treatment of septic shock with the tumor necrosis factor receptor:Fc fusion protein. *The New England Journal of Medicine*.

[21] Clermont G, Bartels J, Kumar R, Constantine G, Vodovotz Y, Chow C (2004). In silico design of clinical trials: a method coming of age. *Critical Care Medicine*.

[22] Wakeland W, Macovsky L, An G A hybrid simulation model for studying acute inammatory response.

[23] NetLogo http://ccl.northwestern.edu/netlogo/.

[24] Painter KJ, Sherratt JA (2003). Modelling the movement of interacting cell populations. *Journal of Theoretical Biology*.

[25] Painter KJ (2009). Continuous models for cell migration in tissues and applications to cell sorting via differential chemotaxis. *Bulletin of Mathematical Biology*.

[26] Goutelle S, Maurin M, Rougier F (2008). The Hill equation: a review of its capabilities in pharmacological modelling. *Fundamental and Clinical Pharmacology*.

[27] Painter KJ, Hillen T (2002). Volume-filling and quorum-sensing in models for chemosensitive movement. *Canadian Applied Mathematics Quarterly*.

[28] Byrne HM, Owen MR (2004). A new interpretation of the Keller-Segel model based on multiphase modelling. *Journal of Mathematical Biology*.

[29] Wang ZA (2010). On chemotaxis models with cell population interactions. *Mathematical Modelling of Natural Phenomena*.

[30] Wagner JG (1968). Kinetics of pharmacologic response I. Proposed relationships between response and drug concentration in the intact animal and man. *Journal of Theoretical Biology*.

[31] LeVeque RJ Finite Difference Methods for Ordinary and Partial Differential Equations Steady State and Time Dependent Problems.

[32] Harten A (1997). High resolution schemes for hyperbolic conservation laws. *Journal of Computational Physics*.

[33] Leonard BP (1988). Simple high-accuracy resolution program for convective modelling of discontinuities. *International Journal for Numerical Methods in Fluids*.

[34] Shu CW, Oshert S (1989). Efficient implementation of essentially non-oscillatory shock-capturing schemes, II. *Journal of Computational Physics*.

[35] Sod GA (1978). A survey of several finite difference methods for systems of nonlinear hyperbolic conservation laws. *Journal of Computational Physics*.

[36] Marrocco A (2003). Numerical simulation of chemotactic bacteria aggregation via mixed finite elements. *Mathematical Modelling and Numerical Analysis*.

[37] Filbet F (2006). A finite volume scheme for the Patlak-Keller-Segel chemotaxis model. *Numerische Mathematik*.

[38] Hafez MM, Chattot JJ (2002). *Innovative Methods For Numerical Solution of Partial Differential Equations*.

[39] Li Y, Karlin A, Loike JD, Silverstein SC (2002). A critical concentration of neutrophils is required for effective bacterial killing in suspension. *Proceedings of the National Academy of Sciences of the United States of America*.

[40] De Waal Malefyt R, Abrams J, Bennett B, Figdor CG, De Vries JE (1991). Interleukin 10(IL-10) inhibits cytokine synthesis by human monocytes: an autoregulatory role of IL-10 produced by monocytes. *Journal of Experimental Medicine*.

[41] Oswald IP, Wynn TA, Sher A, James SL (1992). Interleukin 10 inhibits macrophage microbicidal activity by blocking the endogenous production of tumor necrosis factor *α* required as a costimulatory factor for interferon *γ*-induced activation. *Proceedings of the National Academy of Sciences of the United States of America*.

[42] Martich GD, Danner RL, Ceska M, Suffredini AF (1991). Detection of interleukin 8 and tumor necrosis factor in normal humans after intravenous endotoxin: the effect of antiinflammatory agents. *Journal of Experimental Medicine*.

[43] Beers MH, Porter RS, Jones TV (2006). *The Merck Manual*.

[44] Su B, Zhou W, Dorman KS, Jones DE (2009). Mathematical modelling of immune response in tissues. *Computational and Mathematical Methods in Medicine*.

[45] Price TH, Ochs HD, Gershoni-Baruch R, Harlan JM, Etzioni A (1994). In vivo neutrophil and lymphocyte function studies in a patient with leukocyte adhesion deficiency type II. *Blood*.

[46] Kumar R, Clermont G, Vodovotz Y, Chow CC (2004). The dynamics of acute inflammation. *Journal of Theoretical Biology*.

[47] Reynolds A, Rubin J, Clermont G, Day J, Vodovotz Y, Bard Ermentrout G (2006). A reduced mathematical model of the acute inflammatory response: I. Derivation of model and analysis of anti-inflammation. *Journal of Theoretical Biology*.

[48] Andoh A, Takaya H, Saotome T (2000). Cytokine regulation of chemokine (IL-8, MCP-1, and RANTES) gene expression in human pancreatic periacinar myofibroblasts. *Gastroenterology*.

[49] Hanke CW, Higley HR, Jolivette DM, Swanson NA, Stegman SJ (1991). Abscess formation and local necrosis after treatment with Zyderm or Zyplast Collagen Implant. *Journal of the American Academy of Dermatology*.

[50] MacDonald GA, Greenson JK, DelBuono EA (1997). Mini-microabscess syndrome in liver transplant recipients. *Hepatology*.

[51] Lamps LW, Pinson CW, Raiford DS, Shyr Y, Scott MA, Washington MK (1998). The significance of microabscesses in liver transplant biopsies: a clinicopathological study. *Hepatology*.

[52] Gable AD, Marsee DK, Milner DA, Granter SR (2008). Suppurative inflammation with microabscess and pseudocyst formation is a characteristic histologic manifestation of cutaneous infections with rapid-growing *Mycobacterium* species. *American Journal of Clinical Pathology*.

[53] Lin JC, Chang FY, Fung CP (2010). Do neutrophils play a role in establishing liver abscesses and distant metastases caused by *Klebsiella pneumoniae*?. *PLoS ONE*.

[54] Alsaif HS, Venkatesh SK, Chan DSG, Archuleta S (2011). CT appearance of pyogenic liver abscesses caused by *Klebsiella pneumoniae*. *Radiology*.

[55] Rocha P, Pigozzo A, Quintela B, Macedo G, Santos R, Lobosco M (2012). Modelling the innate immune system. *Bio-Inspired Computational Algorithms and their Applications*.

[56] Lardner A (2001). The effects of extracellular pH on immune function. *Journal of Leukocyte Biology*.

[57] Amento EP, Ehsani N, Palmer H, Libby P (1991). Cytokines and growth factors positively and negatively regulate interstitial collagen gene expression in human vascular smooth muscle cells. *Arteriosclerosis and Thrombosis*.

[58] Duncan MR, Frazier KS, Abramson S (1999). Connective tissue growth factor mediates transforming growth factor *β*- induced collagen synthesis: downregulation by cAMP. *The FASEB Journal*.

[59] Newby AC, Zaltsman AB (1999). Fibrous cap formation or destruction—the critical importance of vascular smooth muscle cell proliferation, migration and matrix formation. *Cardiovascular Research*.

